# CD97/ADGRE5 Inhibits LPS Induced NF-*κ*B Activation through PPAR-*γ* Upregulation in Macrophages

**DOI:** 10.1155/2016/1605948

**Published:** 2016-02-21

**Authors:** Shuai Wang, Zewei Sun, Wenting Zhao, Zhen Wang, Mingjie Wu, Yanyun Pan, Hui Yan, Jianhua Zhu

**Affiliations:** Department of Cardiology, First Affiliated Hospital, School of Medicine, Zhejiang University, Hangzhou 310000, China

## Abstract

CD97/ADGRE5 protein is predominantly expressed on leukocytes and belongs to the EGF-TM7 receptors family. It mediates granulocytes accumulation in the inflammatory tissues and is involved in firm adhesion of PMNC on activated endothelial cells. There have not been any studies exploring the role of CD97 in LPS induced NF-*κ*B activation in macrophages. Therefore, we first measured the CD97 expression in LPS treated human primary macrophages and subsequently analyzed the levels of inflammatory factor TNF-*α* and transcription factor NF-*κ*B in these macrophages that have been manipulated with either CD97 knockdown or overexpression. We found that a reported anti-inflammatory transcription factor, PPAR-*γ*, was involved in the CD97 mediated NF-*κ*B suppression. Furthermore, by immunofluorescence staining, we established that CD97 overexpression not only inhibited LPS induced p65 expression in the nucleus but also promoted the PPAR-*γ* expression. Moreover, using CD97 knockout THP-1 cells, we further demonstrated that CD97 promoted PPAR-*γ* expression and decreased LPS induced NF-*κ*B activation. In conclusion, CD97 plays a negative role in LPS induced NF-*κ*B activation and TNF-*α* secretion, partly through PPAR-*γ* upregulation.

## 1. Introduction

Lipopolysaccharide (LPS) mediated immune-inflammatory response plays an important role in the disease resistance when the body encounters a gram-negative bacterial infection. During this, macrophages, among the multiple immune cells, first help in endocytosis of bacterium debris followed by expansion of local inflammatory response and eventually presenting the antigen to T cells in a MHC class II-dependent manner. This subsequently gives rise to T cell activation and the development of an adaptive innate immune response to clean up the pathogen infection [1–4].

CD97/ADGRE5 is a membrane protein of the epidermal growth factor-seven transmembrane family (EGF-TM7) that belongs to adhesion G protein-coupled receptors (GPCR) [5–7]. It includes three isoforms (EGF1, 2, 5 EGF1, 2, 3, 5 and EGF1, 2, 3, 4, 5) [8–10]. CD97 is widely expressed on the cell surface of lymphoid cells and smooth muscle cells as well as macrophages [11–13]. In tumor, CD97 is highly correlated with invasion and dedifferentiation [14–16]. Moreover, CD97 has also been found to be induced by GM-CSF. Besides, a higher expression of CD97 was found in lipid-laden macrophages of atheromatous plaques [17]. Veninga et al. have showed that CD97 also participated in granulocytes accumulation during acute inflammation [10]. In addition, CD97 also had been suggested to induce the inflammatory response by promoting leukocytes adhesion to the endothelium [18]. Since the CD97 isoform mainly expressed in macrophages is CD97 (EGF1, 2, 5) [8], we planned to verify whether and how direct manipulation of CD97 (EGF1, 2, 5) can regulate NF-*κ*B activation in macrophages. Therefore, we first tested the changes in CD97 expression under LPS treatment and then tried to investigate the relationship between CD97 and NF-*κ*B activation.

## 2. Materials and Methods

The usage of human peripheral blood in this study was according to the principles of Declaration of Helsinki and the appropriate consent from the healthy donors was obtained.

### 2.1. Cell Culture

Human primary macrophages were differentiated from separated monocytes from the blood of healthy adults using lymphocyte separation kit (Sigma, USA). Isolated monocytes were cultured in dishes with RPMI1640 medium (Gibco, USA) containing 10% fetal calf serum (Sigma, USA) and GM-CSF (100 ng/mL) (Peprotech, USA) for 7 days, until they were differentiated into human primary macrophages. The culture medium was replaced with fresh complete medium including GM-CSF every other day. After 7 days, cells were seeded into 6-well plates for the subsequent studies. LPS was purchased from Sigma (USA). The duration of LPS treatment in all experiments was 24 h except the case when indicated time was shown.

### 2.2. siRNA or Plasmid Transfection

The siRNA or plasmid transfections were performed using lipofectamine 3000 transfection reagent (Lifetech, USA) according to manufacturer's instructions. In brief, 2 *μ*L of siRNA (50 *μ*M) (Baiao, China) or 2 *μ*g of plasmid cDNA (Origene, USA) was mixed with 5 *μ*L of P3000 reagent and 3.75 *μ*L of lipofectamine 3000 reagent in a 200 *μ*L Opti-MEM (Gibco, USA) culture medium. After incubation for 5 minutes, mixture was added to the 1 × 10^6^ cells already plated in a six-well plate. Cells were cultured for another 24 hours and later collected for further experiments.

### 2.3. Western Blot

The cells were collected and subsequently lysed with lysis buffer (CST, USA) to extract total proteins. The 40 *μ*g of proteins was loaded onto a 10% polyacrylamide-SDS gel for separation. Next, the proteins were transferred onto PVDF membrane (Millipore, USA) and the membrane was then blocked by incubating with a 5% nonfat milk for 1 hour at room temperature. Later, membrane was hybridized with primary antibody at 4°C overnight followed by washing with TBST for a total of 30 min, three times. Subsequently, membrane was incubated with corresponding secondary antibody for 1 hour at room temperature. Finally, chemiluminescence was detected using ECL kit (Pierce, USA). The antibodies and their dilutions used were as follows: rabbit anti-CD97 (1 : 1000) (Abcam, USA); rabbit anti-GAPDH (1 : 1000) (Nuoyang, China); mouse anti-p65 (1 : 1000) (CST, USA); rabbit anti-PPAR-*γ* (1 : 1000) (CST, USA); rabbit anti-Lamin B (1 : 1000) (Nuoyang, China); goat anti-rabbit (1 : 5000) (Nuoyang, China); goat anti-mouse (1 : 5000) (Nuoyang, China).

### 2.4. Flow Cytometry

Macrophages were treated with LPS (from 0 *μ*g/mL to 60 *μ*g/mL) for 24 h. After being washed twice with phosphate buffer saline, cells were incubated with FITC-conjugated-anti-CD97 (BD, USA). Then, analysis was performed using a flow cytometer (Beckman, USA) with cell Quest Pro Software.

### 2.5. RNA Extraction and qRT-PCR

Total RNA was isolated using the RNA simple total RNA kit (Tiangen, China). The cDNA was synthesized using a primescript RT reagent kit (Takara, Japan). qRT-PCR consisted of 40 cycles of 95°C for 5 s, 60°C for 30 s. 18s rRNA served as an endogenous control. Primers were as follows: CD97-EGF(1, 2, 5): forward (ACTCTGCCGGGAGCTGAAAC) and reverse (TGGATGGTGACCTCGGCTGA); CD97-EGF(1, 2, 3, 5): forward (CGACGCATGTCCATGTCATTTGTT) and reverse (TGCCAAGCTTGTGTTCTAAGT); CD97-EGF(1, 2, 3, 4, 5): forward (CGGCACCACATCCCACATC) and reverse (GGGACCATAAGAGTGATCAAG); 18s rRNA: forward (CCGCACTTGATACGGTTCCT) and reverse (CCAGGCTGATCTATCCCACTG).

### 2.6. Enzyme-Linked Immunosorbent Assay (ELISA)

1 × 10^6^ macrophage cells were transfected with pCMV-basic or pCMV-CD97 plasmids for 24 hours. Later, the fresh culture medium containing LPS (40 ng/mL) (Sigma, USA) was added for another 24 hours. Finally, culture supernatant was collected for detecting TNF-*α* or total protein using an TNF-*α* ELISA kit (RD assays, USA) or a TP (total protein) ELISA kit (Lianke, China), respectively, according to the manufacturer's instructions. Relative expression of TNF-*α* was obtained by normalizing to total protein concentration.

### 2.7. Immunofluorescence

The macrophages (5 × 10^5^) were seeded in the glass bottom of cell culture dish (NEST, USA). After required treatments, cells were first fixed in a fixing solution containing 50% acetone and 50% alcohol and then permeabilized by 0.5% Triton X-100. Next, the cells were incubated with anti-CD97, anti-PPAR-*γ*, or anti-p65 antibodies (1 : 100) at 37°C for 1 hour followed by washings with PBS for 15 minutes. Later, the incubation with goat anti-rabbit FITC IgG or goat anti-mouse rhodamine IgG (Kangwei, China) (1 : 100) was performed at 37°C for 1 hour in the dark. This was followed by DAPI (Gugebio, China) staining and finally the fluorescent signal was observed using Zeiss Confocal Imaging System (Zeiss, Germany).

### 2.8. Electrophoretic Mobility Shift Assay (EMSA)

Nuclear protein extraction and EMSA were performed as described previously [19]. The oligonucleotides containing the NF-*κ*B binding sequence were obtained from Signosis (USA). These oligonucleotides (1.25 pmol) were end-labeled by HRP and later these labeled oligonucleotides (7.5 fmol) were used in the DNA-binding reaction containing 5 *μ*g nuclear extract. The binding reaction also contained 5x binding buffer poly-d(I-C) and ddH_2_O (Signosis, USA). The reaction mixtures were incubated for 30 min at room temperature and subsequently separated by electrophoresis in a 6.5%, nondenaturing gel. After the nuclear extract mixtures were separated on gel, they were transferred onto a binding membrane and cross-linking reaction was performed using BioLink DNA Crosslinker (AJ, German). Finally, chemiluminescence signal was detected by ECL kit using ChemiDoc XRS+ system (Bio-Rad, USA). NF-*κ*B-containing oligonucleotide sequence used was as follows:

 5′-GATCCAAGTCCGGGTTTTCCCCAACC-3′.

### 2.9. Chromatin Immunoprecipitation (ChIP)

The ChIP assay was performed using a kit from Cell Signaling Technology (USA). In brief, 5 × 10^6^ macrophages were fixed with 1% formaldehyde (Aladdin, USA) for 10 min at room temperature. Chromatin DNA was subsequently sheared by micrococcal nuclease (CST, USA) to lengths ranging from 300 to 900 bp at 37°C for 20 min with rotation. The samples were then incubated with rabbit anti-p65 monoclonal antibody (2 *μ*g) or rabbit IgG antibody (2 *μ*g) overnight at 4°C with rotation. The samples were then immunoprecipitated by incubating with protein A/G agarose beads, for 2 hours at 4°C. Next complexes were reverse-cross-linked at 65°C for 6 hours with NaCl (5 M) treatment. After this, DNA was purified by proteinase K treatment and with the help of DNA purification kit (CST, USA). Finally, qRT-PCR was performed to detect the corresponding DNA content. The primers designed for amplification of NF-*κ*B responsive elements in the promoter of TNF-*α* gene [20, 21] were as follows:

 F: TAGCAGAGAGTTGGCTACACACC; R: ACGGCTTCGACCATCAAGTTC.

### 2.10. Generation of CD97-Cas 9 THP-1 Cell Line

The CD97 knockout in THP-1 cells was performed using CRISPR/Cas 9 system according to previous protocol [22]. In brief, gRNA for CD97 was designed and cloned into Pep-ko (Pep-330x) plasmid. After transfection of this plasmid, THP-1 cells were screened/selected using puromycin (2 *μ*g/mL). The surviving cells were further seeded into 96-well plate to culture into monoclonal cell lines for detecting CD97 expression. The sequences for CD97 gRNAs were as follows:

 F-caccgTCCGGTGGACGAGGCGGCGG; R-aaacCGGCCGACCACCACCGCTTc.

### 2.11. Statistical Analysis

All the significant differences between the samples were analyzed using Graphpad prism 5.0 software. The data were presented as the mean ± standard deviations (SD). Comparisons were performed using Student's test. A *p* value of <0.05 was considered to be statistically significant. All experiment was performed independently at least three times.

## 3. Results

### 3.1. CD97 Inhibits TNF-*α* Secretion in LPS Induced Macrophages

**Figure 1:**
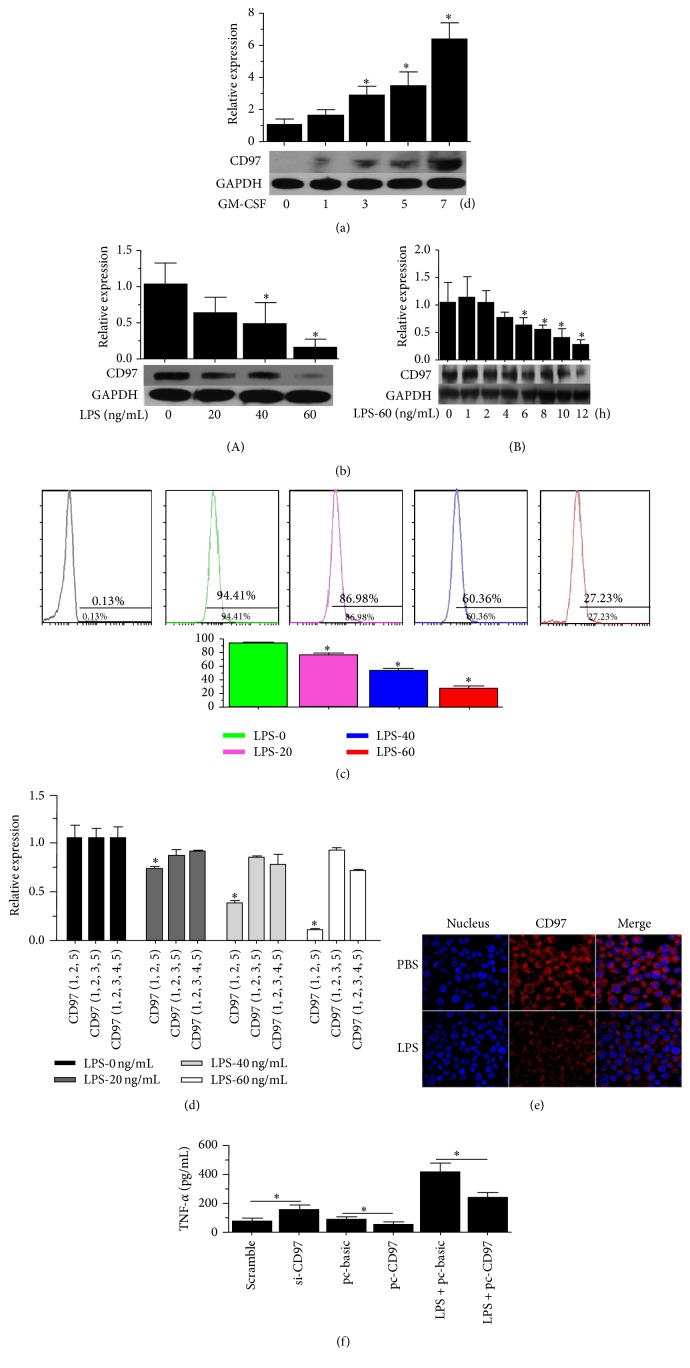
Effect of CD97 on LPS mediated TNF-*α* secretion in macrophages. (a) CD97 protein levels increased when monocytes differentiated into macrophages in the presence of GM-CSF (100 ng/mL). (b) LPS (0 to 60 ng/mL) treatment for 24 h inhibited CD97 protein expression in a concentration dependent manner (A). LPS (60 ng/mL) inhibited CD97 protein levels in a time gradient manner (0 to 12 h) (B). (c) Macrophages were treated with LPS (0 to 60 ng/mL) for 24 h. Cells were incubated with FITC-conjugated-anti-CD97 antibody. CD97 expression was analyzed by flow cytometry. (d) Macrophages were treated with LPS (0 to 60 ng/mL) for 24 h; total RNA was extracted and reverse transcription was performed. Finally, qRT-PCRs were performed to measure the expression of three CD97 isoforms. (e) CD97 expression/distribution in macrophages treated with or without LPS (40 ng/mL) for 24 hours by confocal microscopy. The red color represents CD97 staining. (f) siRNA of CD97 transfection upregulated TNF-*α* secretion whereas ectopic expression of CD97 downregulated TNF-*α* secretion. LPS (40 ng/mL) induced TNF-*α* secretion was blocked by CD97 ectopic expression (*p* < 0.05).

First, we analyzed the expression of CD97 during the process of differentiation from monocytes to macrophages following GM-CSF (human) treatment. We observed that CD97 expression gradually increased and fully differentiated macrophages after day 7 had the highest expression as shown in Figure 1(a). Our data is consistent with the previous published study [17]. In contrast, when we treated these fully differentiated macrophages with different concentrations of LPS for 24 h, we observed a gradual decrease in CD97 expression in concentration (0–60 ng/mL) dependent manner as shown in Figure 1(b)(A). And the CD97 expression was also decreased following the time (0–12 h) gradient manner of 60 ng/mL LPS treatment (Figure 1(b)(B)). In addition, we verified this effect by flow cytometry and immunofluorescence staining. We observed that CD97 expression is indeed reduced (Figures 1(c) and 1(e)). The influence of LPS on the transcriptional level of CD97 was also analyzed. As shown in Figure 1(d), the most abundant isoform of CD97 expressed in macrophages was CD97 (EGF1, 2, 5), and a gradual decrease in CD97 (EGF1, 2, 5) was observed in concentration (0–60 ng/mL) dependent manner of LPS treatment. Further, we analyzed the effect of CD97 (EGF1, 2, 5) expression on TNF-*α* secretion in macrophages. To do this, we transfected the macrophages with CD97 (EGF1, 2, 5) ectopic expression plasmid or siRNA, respectively. As seen in Figure 1(f), CD97 knockdown enhanced TNF-*α* secretion (lane 2), whereas CD97 overexpression exogenously decreased TNF-*α* secretion (lane 4). Moreover, CD97 also reversed the promotion effect of LPS on TNF-*α* secretion (lane 6). This data suggested that CD97 inhibited the LPS induced TNF-*α* secretion.

### 3.2. CD97 Suppresses NF-*κ*B Activity and Its Nuclear Translocation

**Figure 2:**
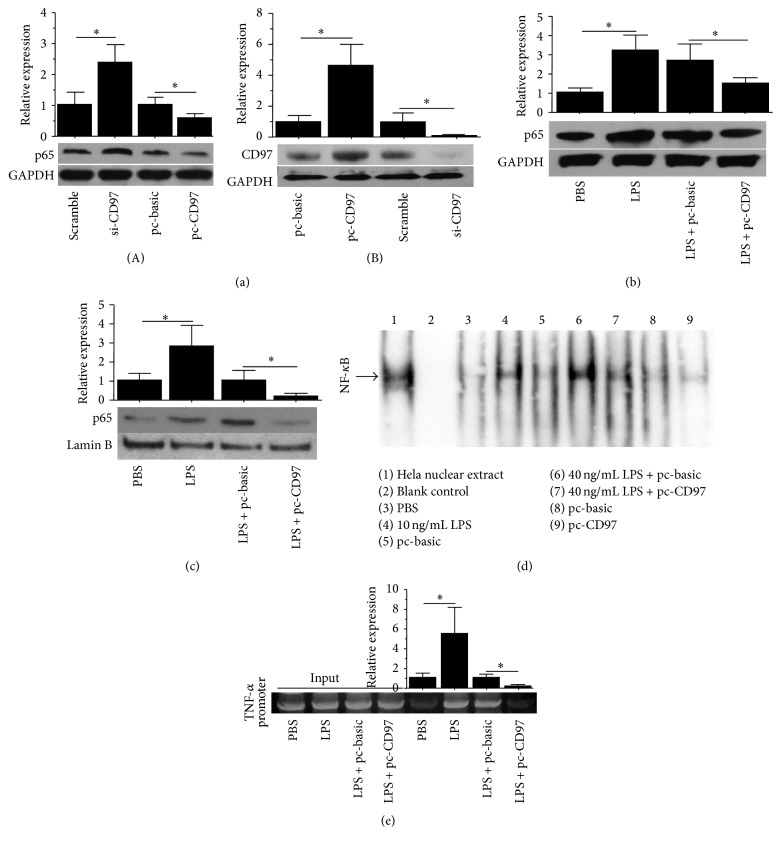
Regulation of LPS induced NF-*κ*B activation by CD97 in macrophages. (a) p65 protein level was increased by siRNA mediated CD97 ablation, while p65 expression was inhibited by CD97 ectopic expression (A). CD97 siRNA inhibited CD97 protein level while its ectopic expression increased it (B). (b) CD97 ectopic expression reversed LPS (40 ng/mL) induced p65 expression. (c) CD97 ectopic expression reversed LPS (40 ng/mL) induced p65 nuclear translocation. (d) Different treatments including LPS concentrations and CD97 overexpression modulated NF-*κ*B nuclear activities as analyzed by EMSA. Lane 1: Hela nuclear extract group (positive control). Lane 2: blank control group. Lanes 3 and 4: LPS stimulation promoted NF-*κ*B translocation. Lanes 5, 6, and 7: CD97 overexpression inhibited LPS induced NF-*κ*B translocation. Lanes 8 and 9: CD97 overexpression inhibited the basal NF-*κ*B nuclear level. (e) ChIP assays were performed with LPS stimulation (40 ng/mL) or LPS stimulation and pc-CD97 transfection using antibodies against p65. Chromatin collected before immunoprecipitation served as an input control. The corresponding NF-*κ*B responsive elements in the promoter of TNF-*α* gene were amplified and analyzed in later qRT-PCRs (*p* < 0.05).

To further understand the role of CD97 in LPS induced TNF-*α* secretion, we focused on NF-*κ*B, a transcription factor which is usually responsible for LPS induced TNF-*α* secretion [23, 24]. As shown in Figure 2(a)(A), ectopic expression of CD97 inhibited the expression of p65, while CD97 knockdown enhanced p65 expression. In addition, CD97 overexpression also inhibited the LPS induced p65 expression (Figure 2(b)). This effect of CD97 was similar to that which we have seen earlier on LPS induced TNF-*α* secretion. To test whether CD97 influenced p65 nuclear translocation, nucleoprotein was extracted. As shown in Figure 2(c), LPS stimulation enhanced p65 nuclear translocation and this effect was reversed by CD97 overexpression. Moreover, through EMSA study, we further confirmed that CD97 upregulation resulted in a marked decrease of NF-*κ*B nuclear translocation in macrophages with or without LPS treatment (Figure 2(d)). To examine whether CD97 influenced the binding activity of p65 on TNF-*α* promoter, ChIP was performed. As shown in Figure 2(e), LPS stimulation enhanced the binding activity of p65 to the TNF-*α* promoter while CD97 overexpression reversed it.

### 3.3. CD97 Reduces LPS Induced NF-*κ*B Activation through Promoting PPAR-*γ* Nuclear Translocation

**Figure 3:**
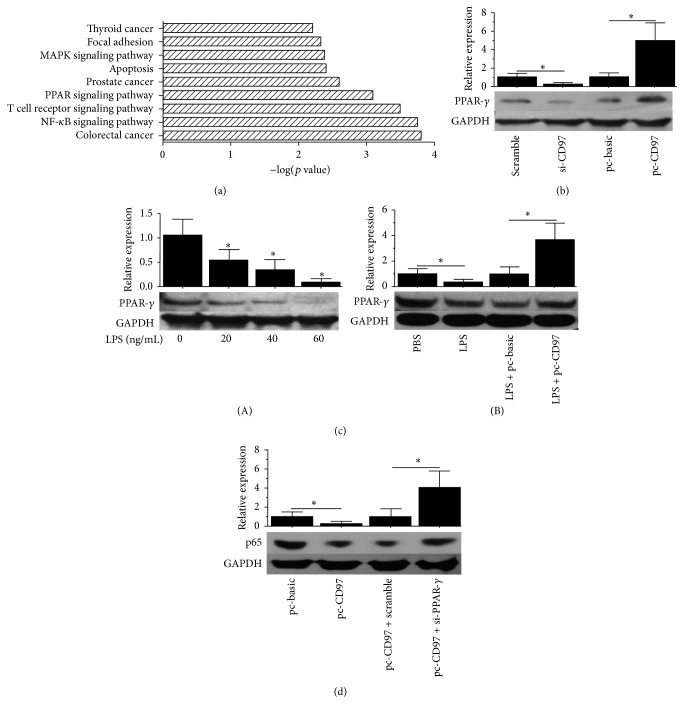
Effect of CD97 manipulations on p65 through increasing PPAR-*γ* expression. (a) Pathways analysis when macrophages were transfected with CD97 overexpression plasmids. (b) CD97 ablation inhibited PPAR-*γ* protein level while its ectopic expression increased it. (c) LPS inhibited PPAR-*γ* protein levels in a concentration dependent gradient (from 0 to 60 ng/mL) (A). CD97 ectopic expression rescued LPS induced PPAR-*γ* inhibition (40 ng/mL) (B). (d) CD97 induced p65 inhibition was rescued by PPAR-*γ* ablation by siRNA.

**Figure 4:**
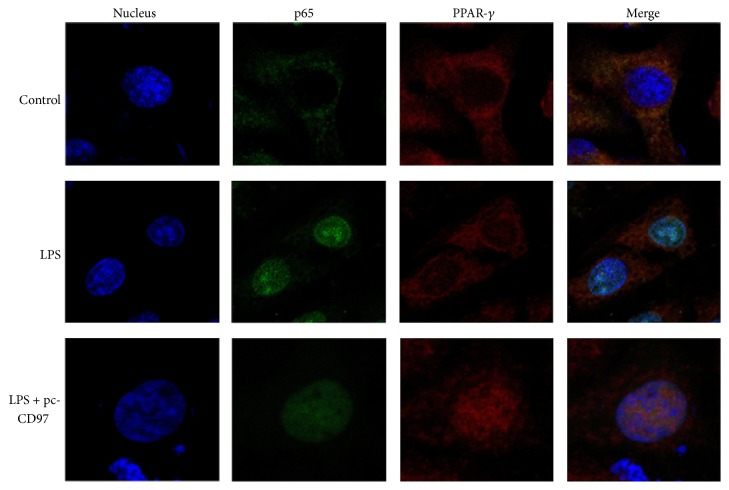
Confocal imaging of p65 and PPAR-*γ* in macrophages. Macrophages were treated with LPS (40 ng/mL) or transfected with CD97 overexpression plasmid and stimulated with LPS (40 ng/mL) for 24 h. Confocal imaging of p65 and PPAR-*γ* was performed. When treated with LPS, p65 displayed nuclear translocation while CD97 overexpression inhibited it but strengthened PPAR-*γ* nuclear translocation. The green signal represented p65 and red signal depicted PPAR-*γ*.

Here we tried to understand the mechanism of how CD97 influenced NF-*κ*B activity. According to our previous ChIP screening data, we assumed that PPAR-*γ*, a widely reported anti-inflammation transcription factor [25–30], played an important role in this effect. We found that PPAR-*γ* expression was highly correlated with CD97 ectopic expression (Figure 3(a)). Therefore, we further investigated the role of PPAR-*γ* and observed that its expression was decreased when CD97 expression was inhibited. Moreover we found that CD97 overexpression increased PPAR-*γ* expression (Figure 3(b)). Besides, a gradual decrease in PPAR-*γ* expression was observed when macrophages were treated with increasing concentrations of LPS (Figure 3(c)(A)). But interestingly the overexpression of CD97 rescued and enhanced the PPAR-*γ* expression as compared to control vector cells in LPS-stimulated macrophages, as shown in Figure 3(c)(B). To further explore the link between PPAR-*γ* and NF-*κ*B, we transfected the macrophages with CD97 plasmid followed by PPAR-*γ* siRNA. As expected, siRNA mediated inhibition of PPAR-*γ* expression reversed the inhibitory effect of CD97 on p65 levels (Figure 3(d)). In addition, we also verified this correlation between p65 and PPAR-*γ* expression/distribution by LPS stimulation in the presence or absence of CD97 by immunofluorescence staining (Figure 4). we found that LPS stimulation enhanced p65 expression in the nucleus where PPAR-*γ* signal was absent. Overexpression of CD97 reversed this distribution by enhancing PPAR-*γ* distribution in the nucleus with decreasing p65 levels. The data here further confirmed that the effects of CD97 on NF-*κ*B nuclear translocation are mediated through PPAR-*γ*.

### 3.4. CD97 Knockout in THP-1 Cells Promotes TNF-*α* Secretion

**Figure 5:**
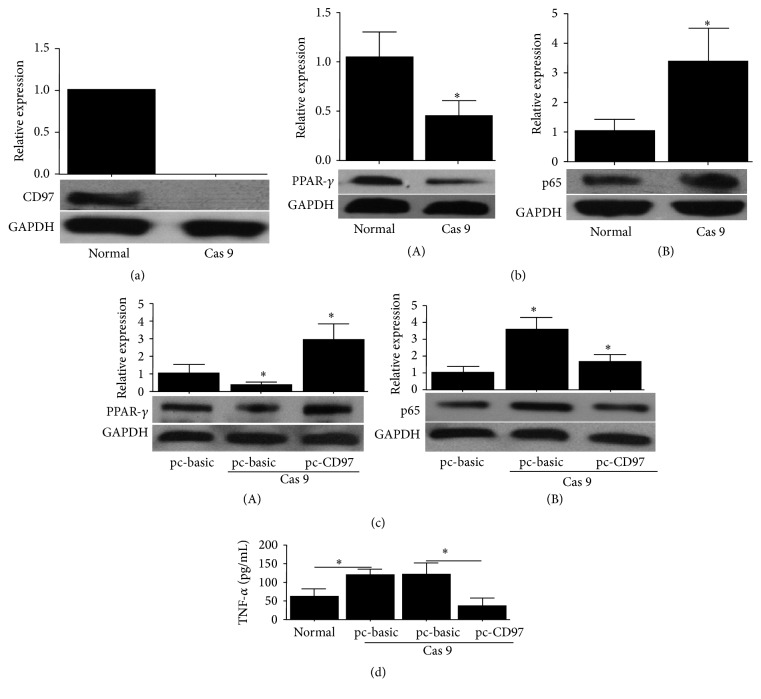
Effect of CD97 knockout on PPAR-*γ* and p65 expression in THP-1 cells. (a) Validation of CD97 knockout in THP-1 cells by CRISPR/Cas 9 system. (b) CD97 knockout inhibited PPAR-*γ* protein level (A), while it increased p65 expression (B). (c) CD97 ectopic expression in CD97 knockout THP-1 cells increased PPAR-*γ* protein level (A) while it inhibited p65 expression (B). (d) TNF-*α* secretion was enhanced in CD97 knockout THP-1 cells while CD97 ectopic expression reversed this effect.

To further reinforce the validity of these results, we also used the latest CRISPR/Cas 9 system to perform CD97 (EGF1, 2, 5 EGF1, 2, 3, 5 and EGF1, 2, 3, 4, 5) knockout THP-1 cells (Figure 5(a)). As expected, the basal expression of PPAR-*γ* was decreased in CD97 knockout cells, whereas p65 expression in these knockout cells was increased (Figure 5(b)). CD97 (EGF1, 2, 5) overexpression in these knockout cells reversed this phenotype with the expression of PPAR-*γ* and p65 increased or decreased, respectively (Figure 5(c)). Finally, the analysis of TNF-*α* levels in these CD97 knockout THP-1 cells revealed that TNF-*α* secretion was increased following CD97 knockout and was inhibited when CD97 (EGF1, 2, 5) was restored by overexpression (Figure 5(d)).

## 4. Discussion

The role of CD97/ADGRE5 in inflammation has been studied extensively [10, 17, 18, 31–33]. These published studies mostly addressed the role of CD97 in leukocyte migration. There is no attempt to study the link between CD97 and NF-*κ*B and also, to the best of our knowledge, the physiological function of CD97 in macrophages cell biology has also not been explored. Therefore, our study was the first to suggest that CD97 (EGF1, 2, 5) plays a negative regulatory role in LPS mediated NF-*κ*B activation and secretion of inflammatory factor TNF-*α* through PPAR-*γ* nuclear translocation. In contrast to other studies on inflammation that involves CD97, it is now amply clear that the influence of CD97 on the inflammatory response is not only through changing leukocytes migration but also through direct suppression of NF-*κ*B activation. In addition we also observed that PPAR-*γ* induced by CD97 inhibited NF-*κ*B activity in macrophages. But to the contrary the work by Thieringer et al. suggested that LPS induced IL-6 and TNF-*α* secretion was not inhibited by PPAR-*γ* activation either in macrophages or in mice model [34]. But there are many other studies consistent with our results. Like telmisartan and activators of PPAR-*γ*, including rosiglitazone and pioglitazone, all inhibited NF-*κ*B activation which was induced by LPS [25–27]. In addition, PPAR-*γ* activation has also been demonstrated to suppress the inflammation in atherosclerosis [34] and inhibit LPS induced NF-*κ*B activation in cardiomyocytes [29]. The inability of PPAR-*γ* activation to inhibit LPS mediated inflammation in Thieringer et al.'s study [34] may be attributed to many factors like the cell status, the drug concentration, and the detection methods or may be due to the contribution of all these factors. For instance, the chemicals used in Thieringer's work were not sufficient or potent enough to completely activate the PPAR-*γ* function. Therefore, our data provides a new insight that CD97 promoted PPAR-*γ* nuclear translocation and negatively regulated the LPS induced inflammatory response. Our work has several limitations. First, the regulatory mechanism of CD97 induced PPAR-*γ* expression needs further investigation. Second, whether PPAR-*γ*'s effects were achieved by regulating the transcription of other genes needed to be further investigated [35]. Third we also need to analyze a CD97-activated anti-inflammatory response.

Therefore, in summary, we conclude that this study is the first to demonstrate that CD97 (EGF1, 2, 5) negatively regulates LPS induced TNF-*α* secretion in monocytes-derived macrophages and this is mediated by CD97 which promoted PPAR-*γ* nuclear translocation and subsequently suppressed NF-*κ*B activation.
